# New Progress in Angiogenesis Therapy of Cardiovascular Disease by Ultrasound Targeted Microbubble Destruction

**DOI:** 10.1155/2014/872984

**Published:** 2014-05-12

**Authors:** Yang-Ying Liao, Zhi-Yi Chen, Yi-Xiang Wang, Yan Lin, Feng Yang, Qiu-Lan Zhou

**Affiliations:** ^1^Laboratory of Ultrasound Molecular Imaging, Department of Ultrasound Medicine, The Third Affiliated Hospital of Guangzhou Medical University, Guangzhou 510150, China; ^2^Department of Imaging and Interventional Radiology, Prince of Wales Hospital, The Chinese University of Hong Kong, Shatin, New Territories, Hong Kong

## Abstract

Angiogenesis plays a vital part in the pathogenesis and treatment of cardiovascular disease and has become one of the hotspots that are being discussed in the past decades. At present, the promising angiogenesis therapies are gene therapy and stem cell therapy. Besides, a series of studies have shown that the ultrasound targeted microbubble destruction (UTMD) was a novel gene delivery system, due to its advantages of noninvasiveness, low immunogenicity and toxicity, repeatability and temporal and spatial target specificity; UTMD has also been used for angiogenesis therapy of cardiovascular disease. In this review, we mainly discuss the combination of UTMD and gene therapy or stem cell therapy which is applied in angiogenesis therapy in recent researches, and outline the future challenges and good prospects of these approaches.

## 1. Introduction

Angiogenesis is a complex blood vessel formation process, involving a variety of angiogenic growth factors synergistic effect. Angiogenesis is divided into two types (physiological and pathological), and the latter always leads to many diseases, such as cardiovascular disease, tumor, and inflammation. Therapeutic angiogenesis, which can improve blood flow, revascularization, and myocardial function, has proved to be one of the most promising therapies for cardiovascular disease. Furthermore, therapeutic angiogenesis has been mainly used for the treatment of ischemic diseases (such as ischemic heart disease). Owing to the disadvantage of invasiveness, limited drug diffusion or lack of selectivity towards targeted tissues, previous drug, or surgical treatment cannot meet the demands of patients and doctors any longer. At the moment, an emerging technique, UTMD, has been proposed for a noninvasive and targeting specific approach in angiogenesis therapy of cardiovascular disease. UTMD refers to that microbubble is exposed to ultrasound (US); on a certain condition, it will be gradually or suddenly activated and/or collapsed. It maybe creates a series of biological effects, including local tissue damage, transient membrane permeability improvement, and extravasation, which are going to facilitate the targeted genes or drugs entering into the tissue or cell of interest [1–4]. UTMD technique has an advantage over other gene delivery methods, mainly reflecting in (1) high safety (being low toxicity and immunogenicity compared with viral vectors and getting rid of threatening ionizing radiation), (2) high cost effectiveness and broad availability (compared with other imaging modalities, a high cost effectiveness makes it more acceptable to be used in clinical application), (3) noninvasiveness and repeatability (microbubbles being always administered intravascularly which makes it possible for repeated applications), and (4) high tissue specificity [5, 6] (drugs or targeted genes being selectively delivered to the only region of interest, rather than nontargeted position). In a word, there are so many advantages that make UTMD a good alternative in angiogenesis therapy of cardiovascular disease.

## 2. The Proposed Mechanism of UTMD

**Figure 1:**
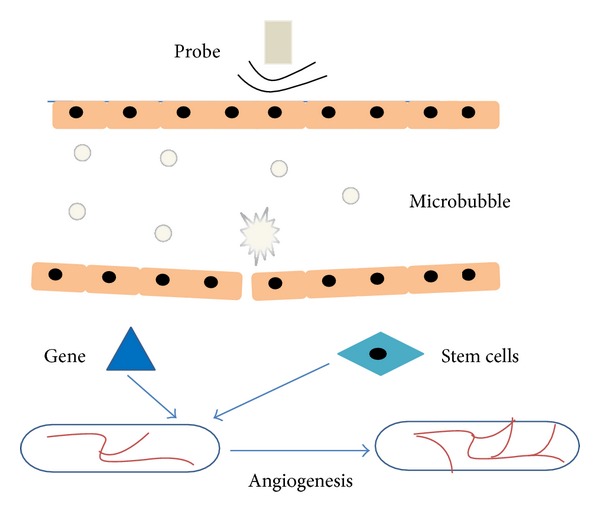
Schematic illustration of UTMD inducing angiogenesis therapy. With the aid of UTMD, targeted genes or stem cells can be delivered to ischemic tissue or organ to promote angiogenesis and increase flow perfusion.

UTMD stands for a technique that molecular bioactive substance or therapeutic gene, injected or incorporated into microbubble in blood circulation, can be eventually released into the targeted tissue or organ under the action of ultrasound. The biological effects produced by ultrasound are used to facilitate gene transfection into targeted tissues or cells; thus, the purpose of targeted therapy is successfully achieved [7]. The commonly used microbubbles, being about 1–10 *μ*m in diameter, can smoothly go through the capillaries, but cannot reach targeted tissue through endothelial gap. Consequently, UTMD mediated gene therapy is mainly based on the biological effects which were produced by the interaction among ultrasound, cell membrane, and endothelial cells. Ultrasound can promote gene-loaded microbubbles local accumulation in lesions, and the biological effects of ultrasound can improve the permeability of the blood vessels and cell membrane, promoting gene exosmosis to targeted lesion and improving the therapeutic effect. These biological effects mainly include the cavitation effect and sonoporation effect. Cavitation effect refers to the cavitation nucleus that is existing in the liquid, after ultrasound exposure, and can produce the dynamic processes mainly including oscillation, collapse, expansion, and contraction [8]. Microbubble can be used as a kind of man-made cavitation nuclei. After injection of microbubble, the concentration of cavitation nucleus in the blood will be increased, which maybe reduces the cavitation threshold, finally enhancing the ultrasonic cavitation effect. Cavitation effect can be divided into instantaneous cavitation and steady-state cavitation. It is generally believed that ultrasound cavitation effect is produced by the following three different mechanisms [9], including (1) producing transient pores on vascular endothelial cell surface to promote macromolecular absorption into the cells; (2) destroying the integrity of the vascular endothelium to make large molecules transfer through the intercellular transfer; and (3) stimulating cell endocytosis function to enhance the intracellular delivery [10]. Sonoporation is described as the phenomenon of forming temporary small holes on cell membrane after ultrasound exposure, which can promote the uptake of extracellular substance into the cell [11, 12]. Numerous studies have showed that sonoporation effect was related to microstreaming, microjet, and shock wave, especially the inertial cavitation, after ultrasound exposure. Moreover, sonoporation has long been regarded as the main mechanism of improving delivery efficiency and it is necessary to optimize sonoporation protocols to obtain a higher transfection rate [13]. In fact, the precise and underlying mechanism of UTMD mediated gene transport in vivo has not been very clear. However, based on what we have known at present, UTMD can be well applied to angiogenic therapy. With the aid of the proposed mechanism of UTMD (such as cavitation effect and sonoporation effect), targeted genes or stem cells can be delivered to ischemic myocardium to promote angiogenesis and restore flow perfusion (Figure 1). Along with the development of mechanism, UTMD technology will play a more and more important role in angiogenesis therapy.

## 3. Gene Therapy

With the rapid development of molecular biology techniques, gene therapy is developing as a novel and promising angiogenesis therapy. Among the researches of gene therapy for cardiovascular diseases, the ischemic heart model is more commonly used. Although the basic research on gene therapy showed the tempting prospects, the results in most of the clinical trial did not achieve the goal of effective treatments, mainly due to the low gene transfection rate and lack of safety [14]. For this reason, there is a great need for designing a safe, efficient, and noninvasive gene delivery approach, which has become a crucial problem to be solved in the gene therapy research. Some researchers began to focus on the safe and noninvasive ultrasound and microbubble. Microbubbles, firstly only developed for clinical diagnostic applications, are now well admitted as nonviral vectors for gene therapy [15]. When they were exposed to ultrasound, microbubbles could strengthen the cavitation effect and facilitate targeted gene delivery [16]. To date, UTMD has been explored as an innovative gene delivery strategy and attracted the attention of many researchers [17]. During the last decade, many efforts and studies have focused on exploring the feasibility of UTMD in angiogenesis therapy and demonstrated that UTMD was a safe and effective targeted transfection strategy for angiogenesis therapy in myocardial ischemia and limb ischemia animal models. The following research was a typical example. In hind-limb ischemia mouse models, Chappell et al. [18] prepared nanoparticles which associated with the gene encoding fibroblast growth factor-2 and injected them into the mouse adductor muscles in the presence of 1 MHz ultrasound. Two weeks later, they observed arteriolar caliber and density were apparently improved in fibroblast growth factor-2 treated group.

### 3.1. Growth Factors Gene Therapy

There are an increasing number of genes for therapeutic angiogenesis, although what are commonly used are still proangiogenic growth factors, mainly including vascular endothelial growth factor (VEGF), fibroblast growth factor (FGF), hepatocyte growth factor (HGF), and angiogenin (Ang). Proangiogenic growth factors, endogenous or exogenous, can markedly promote angiogenesis with the aid of UTMD.

#### 3.1.1. VEGF Gene Therapy

**Figure 2:**
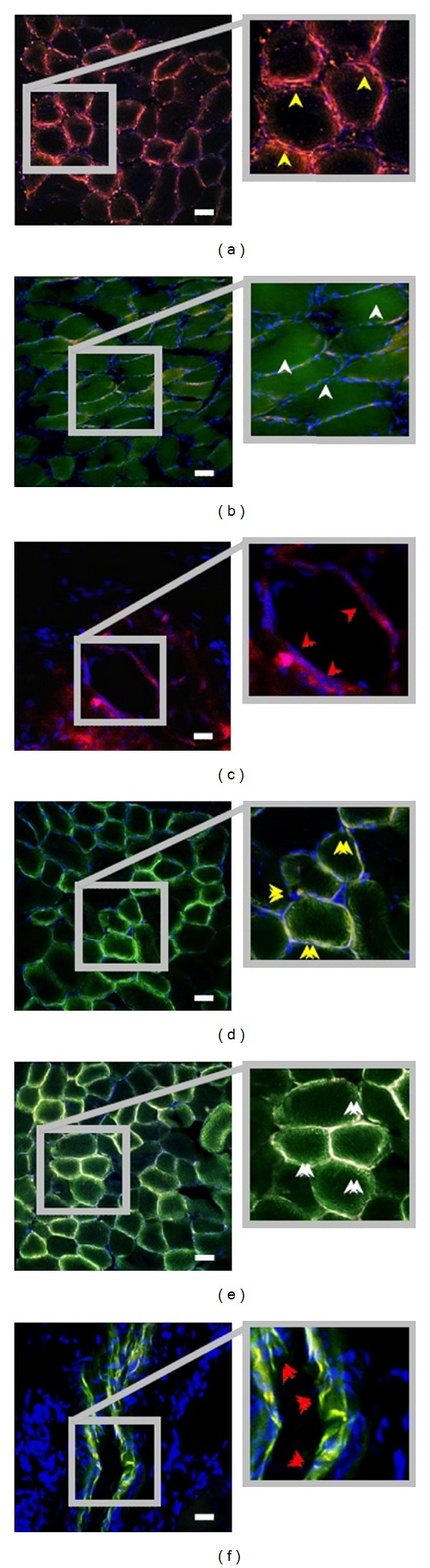
Representative immunofluorescent staining of IM-therapy ((a)–(c)) and UM-therapy ((d)–(f)) ischemic hind-limb muscle, at 17 days following iliac artery ligation, CD31 staining (red); TO-PRO-3-nuclei staining (blue); colocalization of GFP and red CD31 staining (yellow). Within IM-therapy groups: (a) areas without discernable GFP signal (yellow arrows), (b) regions with strong GFP signal (white arrows), and (c) arterioles with little GFP signal (red arrows). In comparison, in UM-therapy groups, a wider diffuse of GFP signal was distributed in (d) both capillaries endothelial (yellow arrows), (e) the adjacent areas (white arrows), and (f) small- to medium-sized arterioles (red arrows). Scale bar = 50 *μ*m. Adapted from [21].

In mammal animals, the VEGF family has five isoforms, including VEGF-A, VEGF-B, VEGF-C, VEGF-D, and placental growth factor [19]. VEGF is necessary throughout the angiogenic process involving vascular endothelial cell migration, infarct size improvement, and apoptosis inhibition. Recent studies show that an increased VEGF expression can promote angiogenic response, under the mechanical effect of UTMD. Taken together, previous studies showed that via increasing the expression of VEGF, the gene therapy could significantly promote angiogenesis and improve cardiac function after myocardial damage [20]. Kobulnik et al. [21] compared the VEGF_165_ gene transfection rate via two delivery methods, ultrasound mediated (UM) of intravenous injection and intramuscular (IM) injection. At 14, 17, 21 and 28 days and 8 weeks following iliac artery ligation, microvascular blood volume (MBV) and microvascular blood flow (MBF) and total transfection efficiency were examined and measured to monitor and evaluate the efficiency of therapeutic angiogenesis. Their results showed that the UM delivery had an advantage over IM delivery in improvement of MBV and MBF, in spite of a lower gene transfection efficiency. This superiority may be related to a wider diffusion and distribution of transgene which was more beneficial to promoting angiogenesis (Figure 2).

**Figure 3:**
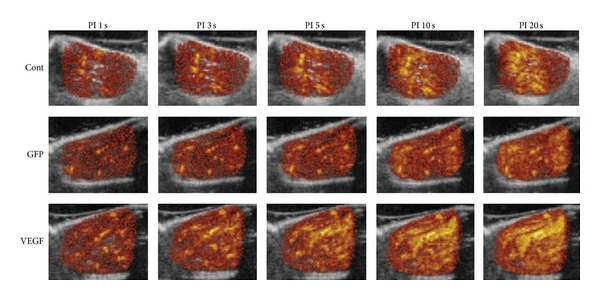
CEU perfusion images of ischemic skeletal muscle were performed at 4 weeks after ligation, showing the corresponding pulsing intervals (PI) versus signal intensity from the animals in the 3 treatment groups. There was a stronger and faster contrast enhancement in VEGF treatment group. Adapted from [22].

Similarly, Leong-Poi et al. [22] conducted a study which was associating ultrasound with microbubbles loaded plasmid DNA encoding the VEGF_165_ in the setting of serious peripheral arterial disease. The results showed that the VEGF_165_/GFP-treated group could significantly enhance the tissue blood perfusion as showed in contrast-enhanced ultrasound (CEU) imaging and remarkably enhance the microvascular density compared with control groups (Figure 3). In this study, the recovery in skeletal muscle perfusion was highly related to the increased noncapillary blood volume, with perfusion peaking at 14 days after transfection. Therefore, they also verified that the UTMD technique held great promise in gene therapy for ischemic disease.

**Figure 4:**
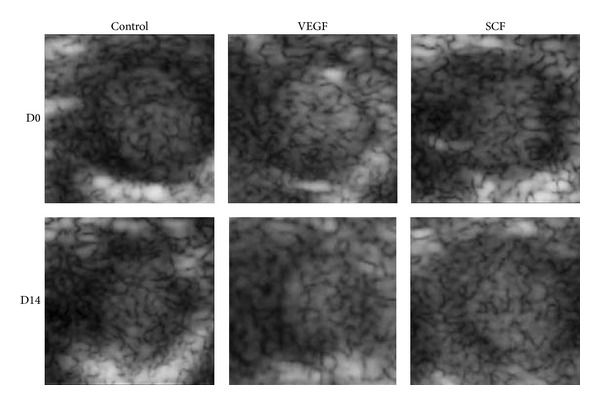
Representative images obtained by myocardial contrast echocardiography performed before (D0) and 14 days (D14) after UTMD mediated empty plasmid (in control group), VEGF, or SCF gene transfection in coronary artery ligation mice. Compared to the control group, blood flow volume was greatly improved in both VEGF and SCF treatment groups. Adapted from [23].

In an animal model of postcoronary artery ligation, Fujii et al. [23] incubated perflutren lipid microbubbles with empty plasmid, plasmid DNA encoding VEGF, stem cell factor (SCF), or GFP. The formative mixture was intravenously administered into the heart which was exposed to Acuson sequoia C256 system (New York, Siemens Medical Solutions Inc) under the ultrasound irradiation (8 MHz, 1.6 mechanical index, 20 min irradiation time, and 500 ms time interval). Two weeks later, they found that GFP was expressed in the infarcted heart, meaning the success of gene transfection. Additionally, vascular density, left ventricular myocardial perfusion, and myocardial function were both observably improved compared with the control group, due to a much higher expression of VEGF and SCF (Figure 4). In consequence, it is hypothesized that the UTMD provides an effective and noninvasive method for delivering targeted gene after myocardial infarction.

#### 3.1.2. FGF Gene Therapy

Asahara et al. [24] were first to demonstrate that FGF gene could facilitate angiogenesis and enhance tissue blood perfusion in a rabbit model of limb ischemia. The major and typical FGF family members, FGF-1 (also called aFGF) and FGF-2 (also called bFGF), are the most commonly used in research. Negishi and his colleagues [25] have delivered the bFGF plasmid DNA and bubble liposomes (BL) into the adductor muscle of the hind-limb ischemia model in the presence of a low intensity of ultrasound exposure. Compared to other treatment groups, a highly efficient gene transfection, the increase of capillary vessels, and quick recovery of the blood flow were observed in the BL with US exposure treatment groups. In this study, UTMD technique was proved to be an effective, noninvasive, and nonviral method in angiogenesis therapy of various ischemic diseases (such as ischemic heart disease). Based on that intramuscular injection could make gene transfection colocalize in the region of the administration site in the previous studies, subsequently, they expanded the previous study for a further step. Therefore, with the aid of US, plasmid DNA, and BLs were systemically administrated into hind-limb ischemia area in the experiment. They found that this method could obtain a more effective and efficient gene transfection and was more suitable for gene therapy of ischemic diseases (such as myocardial infarction) [26]. In a rat model of ischemic myocardium, Zhao et al. [27] elucidated the feasibility of targeting delivery of heparin modified microbubbles (HMB) carrying aFGF into ischemic myocardium with the help of UTMD technique. A remarkably promotion of myocardial vessel neogenesis, which was proved by M-mode echocardiography, contributed to remarkable enhancement of regional and global cardiac functions. As shown in hematoxylin and eosin staining of acute myocardial infarction, ischemic myocardium was significantly improved in aFGF-HMB + US treatment group, being consistent with cardiac function results. This study suggested that cavitation induced by ultrasound and HMB could accelerate FGF gene therapy of ischemic myocardium.

#### 3.1.3. HGF Gene Therapy

Previous studies have shown that HGF played an important part in cell growth and motility and angiogenesis process. Therefore, it may be a better candidate gene for angiogenesis therapy [28]. In a rabbit ischemia model, HGF gene therapy has been firstly proven to effectively promote gene transfection, with the help of UTMD [29]. In 2011 [30] and 2012 [31], Yuan et al. demonstrated that the combinations of an angiogenic gene and UTMD technique could enhance the expression efficiency of the delivered gene. As a consequence, it was identified as a useful angiogenic gene therapy of ischemic heart disease. Along the same line, they had a deeper and further research. In the experiment, they tested the therapeutic effect of combining UTMD with plasmid DNA encoding the HGF in treatment of myocardial infarction. They found that HGF + MB + US treatment group could significantly reduce the infarct size and left ventricle weight, along with augmenting microvessel density, which was consistent with their previous research results [32]. In addition, Zhou et al. [33] have reported that after the UTMD-induced gene delivery, the expression of HGF gene was significantly improved while only a negligible impact on cell viability was observed. At the same time, it could also improve angiogenesis, blood flow, and fibrosis in myocardial ischemia. Therefore, it was identified as a novel and promising gene therapy of cardiovascular disease.

#### 3.1.4. Ang Gene Therapy

Ang is one of much concern cytokines that promote angiogenesis, with its family members mainly including Ang1, Ang2, Ang3, and Ang4. Ang1 and its receptor Tie2 are most widely studied and Ang1/Tie2 system has an effect on angiogenesis in the late stage, predominantly accelerating endothelial cell migration and maintaining his survival. Previous studies have proved that UTMD can significantly improve transfection efficiency of Ang-1 gene both in vitro and in vivo, suggesting that this transfection strategy can be used for angiogenic therapy. Furthermore, in order to enhance the hAng-1 gene transfection efficiency, Zhou et al. [34] explored and tested some transfection parameters (such as FBS, the cell suspension, and adherent mode), no longer limited to ultrasound irradiation parameters in previous studies. The obtained optimized transfection parameters lay the foundation of future research of UTMD assisted angiogenic gene therapy of cardiovascular disease. Meanwhile, they pointed out that we should not blindly pursue the maximal gene transfection rate, while ignoring the cell or tissue damage.

### 3.2. Other Gene Therapies

Except for common angiogenic growth factors, an increasing amount of genes have been studied the potentiality for treatment of cardiovascular disease. Taking the protein kinase Akt as an example; previous studies have showed that Akt could protect heart from damage and restore cardiac function. Therefore, Akt also was regarded as a therapeutic target for gene therapy of cardiovascular disease. Taking into consideration the low transfection rate of the commercially available Definity microbubble; Sun et al. [35] used a cationic microbubble, which has the ability to bear more plasmid DNA, to transfer the Akt targeted gene to the animal model of ischemic myocardium. The measured data showed that the combination of UTMD and Akt therapeutic gene had a bright future in angiogenesis therapy. Moreover, Li et al. [36] also tested the feasibility and safety of exogenous Akt1 gene delivery under the condition of UTMD in the myocardium of new-born gender rats. When several ultrasound parameters were simultaneously optimized, it was possible to improve the efficiency of gene transfection and only produce a minor side reaction. Their study suggested the method of UTMD-assisted exogenous Akt1 gene transfection had a chance to be applied to gene therapy of cardiovascular disease.

**Figure 5:**
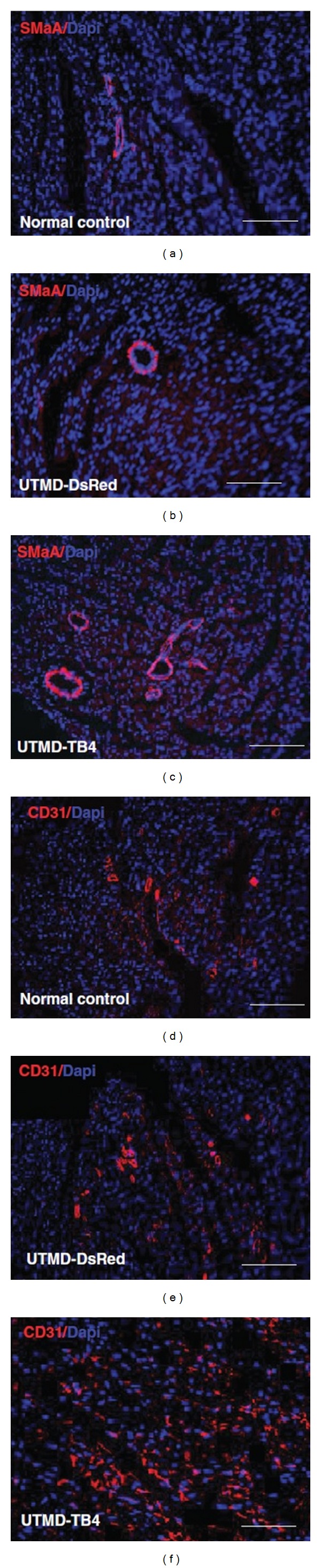
Representative microscopic images of angiogenesis after UTMD mediated TB4 treatment (scale bar = 150 mm). ((a)–(c)) The top panels are stained antibodies against smooth muscle a-actin (SMaA) (red) and nucleus (blue). Groups from left to right are, respectively, normal control, DsRed control, and UTMD-TB4 treated. An increase in SMaA was observed in the UTMD-TB4 treatment group, being consistent with coronary arteriogenesis response. ((d)–(f)) The middle panels are similar except the antibody is against CD31. Adapted from [38].

Additionally, previous study indicated that thymosin beta 4 (TB4) played an important role in facilitating cardiac neovascularization, promoting cell proliferation and differentiation, and maintaining myocardial function following the adult heart ischemic damage [37]. In order to avoid other nontargeted organs affected and obtain a higher and longer targeted gene transfection, Chen et al. [38] combined the piggybac transposon-mediated gene-delivery system with UTMD technique in an experimental study. In their study, they systemically administrated exogenous TB4 gene and then dealt with them under the UTMD. Results showed that adult resident cardiac progenitor cells were induced to proliferate and form three major cardiac lineages. In addition, the treatment groups could dramatically augment coronary artery and capillary density, indicating generating angiogenesis and arteriogenesis (Figure 5). Based on the above results, it is considered as that the therapeutic method could be used for angiogenesis therapy.

### 3.3. Multigene Therapy

**Figure 6:**
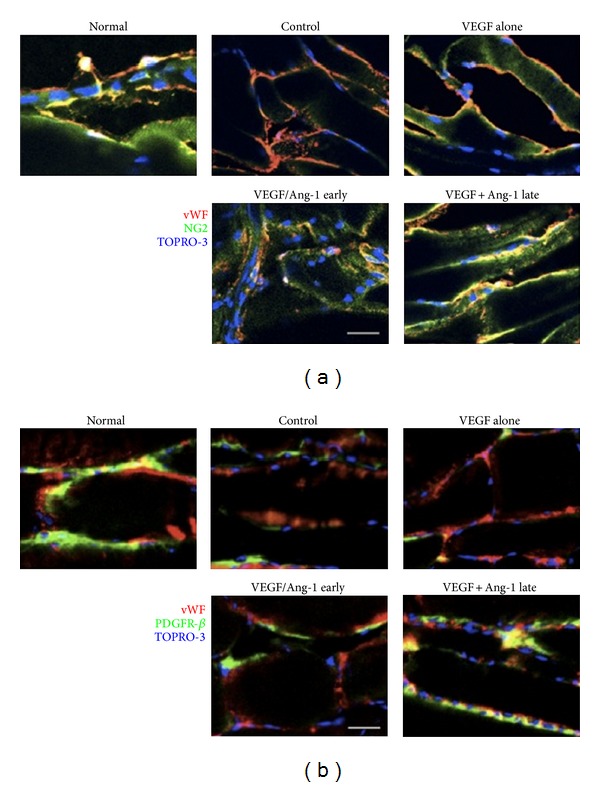
Representative images of pericyte coverage from nonischemic and schematic adductor muscle in respective therapy group at 8 weeks after ligation, von willebrand factor (vWF)-endothelial cells (red), and TOPRO-3-nuclei (blue). (a) NG2-pericyte coverage, (green). Scale bar = 100 *μ*m. (b) Platelet-derived growth factor receptor (PDGFR)-beta-pericyte coverage, (green). Scale bar = 20 *μ*m, colocalization of vWF and either NG2 or PDGFR-beta (yellow). There were the greatest pericytes expressing NG-2 (a) and PDGFR-beta (b) in VEGF + Ang-1 late groups. Adapted from [41].

Angiogenesis is a complex blood vessel formation process, involving a variety of angiogenic growth factors synergistic effect, and it finally comes into being mature vascular beds. Successful therapeutic angiogenesis should be able to enhance their treatment effects as well as alleviate the undesirable impact by complementary effects of several angiogenic growth factors. Some researches on combinations of several growth factors have been explored in animal models of coronary artery disease and peripheral arterial disease. With the aid of viral vectors, a combination gene therapy of VEGF early and Angiopoietin-1 (Ang-1) has been demonstrated that it could promote angiogenesis, facilitate cardiomyocyte proliferation, and decrease apoptosis and ventricular remodeling [39, 40]. Smith et al. [41] designed a similar experiment with the aid of UTMD. In a rat model of chronic hind-limb ischemia, delivering VEGF early and Ang-1 5 min later was performed under the UTMD. VEGF with Ang-1 late delivery groups could increase and/or sustain vessel density, blood flow, and flow reserve, along with enhancing pericyte coverage at 8 weeks after ligation (Figure 6). They also came up with that it was of great importance to imitate the timeline of endogenous gene expression in order to promote angiogenesis.

### 3.4. Assessment of the Efficacy of Gene Therapy

**Figure 7:**
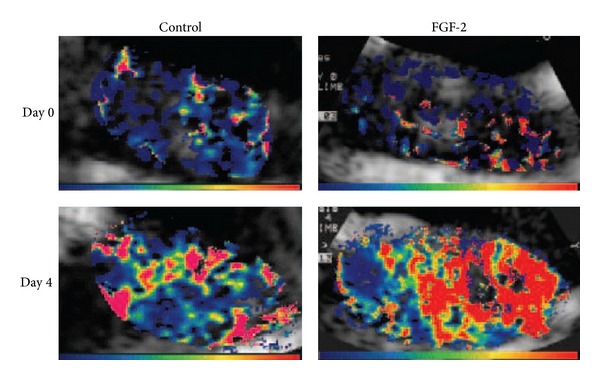
Representative color-coded CEU images reflecting retention fraction of Integrin *α*V*β*3-targeted microbubbles in control and ischemic proximal hind-limb adductor muscles from untreated and FGF-2-treated rats after ligation (Day 0) and at day 4 after ligation (Day 4). Color scales appear at bottom. Adapted from [42].

Gene therapy of angiogenesis has turned out to be a promising method in preclinical studies; however, the therapeutic effect in clinical trials has not reached the target that was expected initially. Therefore, it is necessary to exploit new means to monitor and evaluate the efficacy of gene therapy of angiogenesis. For example, CEU and targeted microbubbles was one of the approaches. What is important, the noninvasive technique not only can detect the safety of some novel therapeutic methods before they get into clinical application, but also can evaluate the treatment effect and guide the next step therapy. The feasibility of this approach was firstly demonstrated by Leong-Poi et al. [42]. In a model of chronic hind-limb ischemia rat, the treated group was injected FGF-2 via intramuscular after iliac artery ligation. They observed and assessed blood flow (by CEU perfusion imaging) and oxygen tension (by phosphor quenching) at 0, 4, 7, 14, or 28 days after ligation. They observed that the signal generated by Integrin *α*V*β*3-targeted microbubble was markedly augmented at 4 days after ligation in FGF-2 treated group, coinciding with a greater angiogenic response (Figure 7). Therefore, they draw a conclusion that CEU and targeted microbubbles could assess gene therapy of angiogenesis. Meanwhile, CEU imaging can provide more information about the pathophysiology of angiogenesis, and it will greatly promote the development of angiogenesis therapy. Therefore, we should take full advantage of this technique in the future, not only in the preclinical but also in clinical trials.

## 4. Stem Cell-Based Therapies

**Figure 8:**
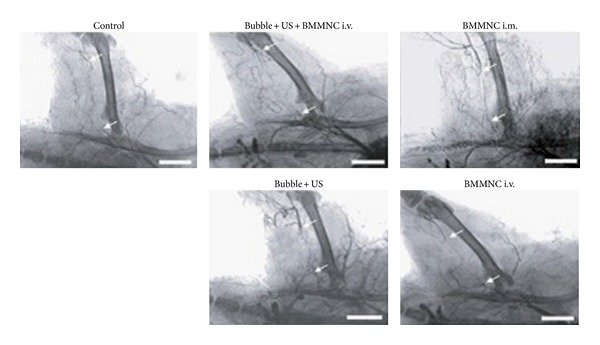
Compared with the BMMNC i.v. Group, the collateral vessel formation in the Bubble + US + BMMNC i.v. and BMMNC i.m. Groups were very obvious, respectively, enhancing 4.2 ± 0.2-fold and 4.3 ± 0.2-fold; the Bubble + US Group was 1.8 ± 0.1-fold, being much less than Bubble + US + BMMNC i.v. Group; Control Group was an equal degree. Adapted from [46].

Stem cells have been proved an alternative angiogenesis therapy approach of cardiovascular diseases (such as peripheral vascular disease and myocardial infarction disease). Bone marrow cell (BMC) is a typical example. In a systematic review, the treatment effect of migrated adult BMC was critically evaluated by meta-analysis, after follow-up of the patients who suffered from ischemic heart disease. It came to a conclusion that the therapeutic benefits were considerable and positive, and this therapeutic method has a prospect on ischemic heart disease [43]. However, the low transplantation efficiency and poor survivability of migrated cells were the major limiting factors in the development and application of stem cell-based therapy [44]. Hence, it is very necessary to improve the poor engraftment rate. Currently, ultrasound and microbubble can also be regarded as a novel and promising gene delivery methods for facilitating drug, gene, and stem cells targeted transportation [45]. Due to spatially and temporally targeting tissues, noninvasive UTMD technology can make sure that stem cell can be selectively delivered into area of interest. Studies have proved the combination of UTMD and stem cell treatment could strengthen the efficacy of transplantation and was a promising method in angiogenesis therapy of cardiovascular disease. In a rat model of ischemic hind-limb, Imada et al. [46] demonstrated that the combination of UTMD and bone marrow mononuclear cells (BMMNCs) method could facilitate angiogenesis and arteriogenesis response. They observed that BMMNCs were transplanted and attached to the endothelium via electron microscopy. In addition, as showed in angiography, newly formed collateral vessels were obviously enhanced in Bubble + US + BMMNC i.v. group, on postoperative day 28 (Figure 8). Therefore, the method of combination of UTMD and BMMNCs maybe has potential applications in myocardial infarction disease treatment.

While they are applied to imaging, microbubbles also can be used as a nonviral vector for inducing stem cell homing [47]. The mechanism of UTMD in promoting stem cell transplantation is possible to be related to sonoporation. The mechanical effect of UTMD could improve myocardial permeability to markedly enhance mesenchymal stem cells (MSC) transplantation to the ischemic myocardium. In addition, with the aid of UTMD, the damaged heart or blood vessels can be stimulated to secrete many kinds of cytokines (such as VCAM-1). Finally, it can accelerate stem cells or progenitor cells attachment and homing, promote angiogenesis, and repair the damaged heart cells [47]. The recent researches of the combination of commonly used stem cells and UTMD in therapeutic angiogenesis are showed in the Table 1.

## 5. Genetically Modified Stem Cell Therapy

**Figure 9:**
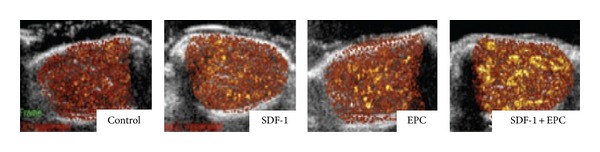
At 2 weeks after iliac artery ligation, all groups were performed on CEU. Above color-coded CEU perfusion images showed ischemic hind-limb muscle blood flow, indicating CEU signal from microbubbles was strongest for combination of SDF-1 and EPC-treated ischemic muscle. Adapted from [50].

Stem cell therapy is identified as a promising and useful treatment method for regenerative medicine. However, the limited proliferation and poor survivability of migrated cells impede their development and application. Recent literatures have reported that genetic modification of stem cells methods could enhance the therapy efficacy in cardiovascular disease. Meanwhile, it came to a conclusion that the stem cell-based gene therapy could overcome the weakness of each other and make both better by complementary of two methods [48]. In previous studies, microbubbles usually served as a nonviral vector to deliver drugs or genes into targeted tissues and cells. Recently, microbubbles have begun to be applied to inducing stem cell-based gene therapy. Otani et al. [49] have reported that UTMD could markedly increase the efficiency of delivering migrated stem cells (MSC and adipose tissue-derived stromal cells) which were genetically modified by small interfering RNA. Despite that this new method maybe produces cell damage, it is better than a viral technique-assistance in the past. Therefore, they came to a conclusion that the novel method had therapeutic potential for cardiovascular diseases in the future. Based on their previous research results, Kuliszewski et al. [50] hypothesized that UTMD in combination with stromal cell-derived factor-1 (SDF-1) had the ability to promote the homing and recruitment of delivered EPCs into chronically ischemic tissue, which proved neovascularization response. In a rat model of hind-limb ischemia, they intravenously administered microbubbles bearing SDF-1 plasmid DNA and exogenous EPCs into the ischemic tissue with the aid of UTMD. At 14 days after delivery, blood perfusion and vascular density in chronically ischemic area were significantly enhanced in the combination of SDF-1 and EPCs treatment group (Figure 9).

## 6. Summary and Prospect

Overall, UTMD mediated gene therapy and stem cell therapy have emerged as novel methods to enhance angiogenesis. So as to get the best curative effect based on UTMD, future studies should go on to explore the UTMD technique to both obtain maximal transfection efficiency and minimal side effect. Here are some questions that are worthy of attention and further research.

### 6.1. Security

Ultrasound was applied into medical treatment since 1930s. Later, the safety of ultrasound in therapeutic application has been also a problem worthy of concern and a complicated research at the same time. A large number of works have reported that the application of ultrasound and microbubbles could cause adverse bioeffects (such as a negative chronotropic effect [51] and renal tissue damage [52]). Most of the current studies of UTMD technology are only in the preclinical stage (either in vitro or in small animal models), and there is still much room for development in the future. Taking into account the security concerns, future research should focus on large animals to continue to repeatedly identify the possible human adverse events before clinical trials begin.

### 6.2. Transfection Efficiency

In the previous research, some investigators have demonstrated that UTMD technique appears to be feasible as a noninvasive and effective approach for angiogenesis therapy. However, there are still many influential factors that limit its application in the clinical treatment. Transfection efficiency is one of very important factors. At present, many researchers have studied the transfection parameters (such as microbubble concentration and composition, the ultrasonic intensity, duty cycle, plasmid DNA concentration, and DNA phosphate ratio) which can dramatically affect the transfection efficiency [34, 53, 54]. And they found that a higher transfection efficiency and a lower cell death could be simultaneously obtained by means of optimizing parameters [55]. Numerous studies have proved that microbubbles could increase the gene transfection efficiency [54, 56]. When microbubbles were exposed to ultrasound irradiation, it would produce a temporary increase of cell membrane permeability, thus promoting gene transfection into the target tissue. However, if the microbubbles concentration is too high or too low, it will be unfavorable for gene transfection. Only under a moderate microbubbles concentration, can be achieved a higher gene transfection, not causing serious cell or tissue damage at the same time. In the future, it is of necessity to explore and build novel microbubbles which will be more beneficial to improve gene transfection.

In the process of UTMD induced gene transfection, with the transfection rate increasing, it is likely to result in a decreased cell viability. For this reason, we should choose the appropriate level of ultrasonic radiation intensity, exposure time, and concentration of microbubbles in future researches. Under such conditions, it is possible to achieve a higher transfection efficiency, as well as ensure a lower cell or tissue damage. There are still many deficiencies in the current research work. What needs to be done in the future is to continue to systematically study and explore the optimization of transfection parameters, laying the theoretical foundation for more effective gene therapy.

Moreover, it is necessary to promote collaboration between UTMD technology and other transfection methods. Believing that with further collaboration among stem cell transplant experts, ultrasound engineers, and cardiovascular biologists, there will be developing more innovative and more mature methods for angiogenesis therapy of cardiovascular disease.

## Figures and Tables

**Table 1:** Summary of experimental studies of UTMD mediated stem cell therapy of angiogenesis.

Author	Candidate stem cell	Animal model	Main finds and results
Tong et al. [57]	BMSCs	Myocardial infarction (rat)	have no effect on the proliferation or apoptosis of MSCs; more efficiently migrate MSCs; a much higher expression of CXCR4, SDF-1 and VEGF; markedly improve the cardiac function and capillary density

Ling et al. [58]	MSCs	Myocardial infarction (mongrel dogs)	markedly improve myocardial perfusion; significantly improve heart function and the wall motion score index; markedly enhance MSC transplantation

Xu et al. [47]	MSC	Myocardial infarction (New Zealand rabbits)	markedly improve the cardiac function; much more capillaries; increase the expression of adhesion molecule and VEGF; enhance the myocardial permeability of microvessel; significantly decrease the area of cardiac fibrosis

Song et al. [59]	BMSC	Myocardial infarction (rabbits)	improve the efficacy of cardiac cell therapy; improve cardiac function in infarcted heart; strengthen the collateral circulation

Kuliszewski et al. [50]	EPC	Chronic hind-limb ischemia (rats)	targeted delivery to the vascular endothelium; greater local engraftment of EPCs; the most obvious improvement in tissue perfusion and capillary density

Ghanem et al. [60]	MSC	After acute myocardial infarction (female wistar rats)	enhance endothelial cell targeting adhesion; augment myocardial engraftment of MSCs

Zhong et al. [61]	MSC	Ischemic myocardium (mongrel dogs)	a much higher expression of various cytokines; provoke inflammatory response and minor myocardial injure; markedly enhance MSCs transplantation to the ischemic myocardium.

^*※*^Mesenchymal stem cells (MSCs), endothelial progenitor cells (EPCs), and bone marrow mesenchymal stem cells (BMSCs).
